# Piloting an AI Introduction Program for Incoming Medical Students: A Novel Approach to Medical Education

**DOI:** 10.1007/s40670-026-02782-9

**Published:** 2026-06-06

**Authors:** Kenny T. L. Ta, Ayush Patel, Nathan Keller, Shivansh R Pandey, Abhinab Kc, Cade Arries

**Affiliations:** University of Minnesota Medical School, Minneapolis, MN 55455 USA

**Keywords:** Artificial intelligence, Undergraduate medical education, Curriculum development, Medical students, Medical ethics

## Abstract

**Background:**

Artificial intelligence (AI) is increasingly recognized for its potential to enhance personalized learning and clinical decision-making in medical education. However, limited integration of AI into medical curricula may leave students feeling underprepared for AI-driven healthcare. This study piloted an AI education program for first-year medical students, assessing post-session perceptions of knowledge, confidence, and perspectives regarding AI in medicine.

**Methods:**

An interactive AI session was conducted for 241 first-year medical students at University of Minnesota Medical School. The session included components of lecture, live demonstrations, and exercises with AI tools such as ChatGPT, Copilot, and Gemini, where students applied AI to various educational and clinical scenarios. Discussions included potential benefits, harms, and limitations including ethical implications, bias and privacy concerns. A post-session survey captured students’ understanding, perceived utility, and confidence regarding AI use in education and in medicine.

**Results:**

Of 241 medical students, 180 attended the session, and 92 of attendees (51%) completed the post-session survey. Findings showed that a majority recognized AI’s value for enhancing study efficiency and diagnostic support, with over 50% indicating increased confidence in incorporating AI tools into their education and possible future practice. Concerns emerged around AI reliability, potential over-reliance, and ethical issues, especially regarding data privacy and bias.

**Conclusions:**

This pilot study provides preliminary evidence supporting the early introduction of AI concepts in medical education. Students’ ethical concerns highlight the need for balanced curricula that combine foundational AI literacy with critical ethical understanding. The study’s descriptive, post-session design limits causal inference; future work should incorporate pre- and post-assessments and longitudinal follow-up to evaluate the sustained impact of AI education on clinical competencies and ethical awareness.

## Practitioner's Points

1. Early and Structured Artificial Intelligence Education is Crucial. With the increasing use of Artificial Intelligence (AI) tools in modern day practice, it is crucial learners are up-to-date with modern practices rather than behind after exiting training. Our pilot program's findings suggest that introducing AI concepts to medical students as early as their first-year orientation may be highly effective. An initial, mandatory session resulted in high post-session students' confidence and perception of AI tools, creating an equitable foundation for all learners, regardless of their prior exposure. This early introduction can help normalize AI as an integral part of modern medicine and prepares students to engage with it throughout their training.

2. Balance Technical "How-To" with Critical and Ethical Discussions. Our post session survey results suggest the duality of learner perception of AI tools. Students are enthusiastic about AI's potential to improve study efficiency and provide diagnostic support, while having significant, valid concerns. The most prominent issues are the accuracy and reliability of AI-generated information, the risk of over-reliance that could degrade clinical skills, and ethical issues like data privacy and algorithmic bias. An effective curriculum must directly address these concerns, teaching students not only how to properly use AI tools but also how to critically evaluate their outputs and use them responsibly.

3. Plan for Longitudinal Integration, Not a One-Off Session. While a single introductory session is a valuable starting point, our study's future directions highlight the need for a longitudinal and integrated curriculum. AI education should not be isolated to an orientation week. To be truly effective, it must be woven into subsequent courses, such as clinical skills and clerkships, with dedicated sessions on clinical applications (e.g., differential diagnosis, documentation) and deeper ethical considerations. This approach reinforces learning and demonstrates AI's practical role in evolving clinical practice.

## Background

The rapid advancements in artificial intelligence (AI) technologies have prompted transformation in various fields, including healthcare and medical education [1]. AI technologies, such as machine learning and natural language processing, have shown promise in various medical applications, including diagnostic support, treatment planning, and patient education [2, 3]. The potential for AI to enhance medical education by providing personalized learning experiences, improving study efficiency, and offering interactive educational tools has been widely recognized [4, 5]. However, the integration of AI into medical curricula remains limited, with many institutions lacking formal AI education programs [6]. As future healthcare providers, medical students should be prepared to engage with these technologies, understanding both their applications and limitations in clinical practice.

Previous studies have highlighted the need for AI education in medical schools, emphasizing the importance of preparing students for the ethical, technical, and clinical challenges posed by AI [5, 7, 8]. Despite the rising need for medical education in AI, there is a paucity of research on medical students’ perceptions and their preferred methods for learning about AI in medicine [6].

Several pedagogical frameworks have inspired the design and rationale of our introductory session. Kolb’s experiential theory postulates that effective learning occurs through a cycle of experience, reflective observation, conceptualization, and experimentation [9]. The session’s structure combining lecture with live demonstrations, hands-on exercises, and large group discussion was designed to engage each phase of Kolb’s experiential theory. Constructivist learning theory further supports this design, emphasizing that learners build knowledge through active engagement and social interaction rather than passive reception [10]. Students were divided into small-group breakout rooms for hands-on activity and ethical discussion, specifically intended to elicit collaborative interaction and problem-solving that will be used in healthcare. Additionally, frameworks of self-regulation suggest providing students with guided exposure to AI tools may support the development of metacognitive strategies for evaluating when and how to use AI effectively [11]. Lastly, critical digital literacy frameworks set the foundation for our session, highlighting the importance of not only merely teaching technical proficiency, but also building the tools to critically evaluate, question, and contextualize digital tools [12]. This competency will be critical as AI continues to grow in medicine and known hallucinations from these tools [13].

These pedagogical frameworks were combined with competency frameworks for AI in medical education set forth by the AAMC focusing on maintaining a human-centered focus; ensuring ethical and transparent use; providing equal access to AI; fostering education, training, and continuing professional development; developing curricula through interdisciplinary collaboration; protect data privacy; and continuous monitoring and evaluation [14].

Through these frameworks, we aimed to evaluate and pilot an AI introduction educational session tailored to incoming medical students, offering a foundational understanding of AI technologies and how they may be used in their academic and professional careers in healthcare. Through a post-session survey, this study assessed students’ self-reported perceptions of AI knowledge, their views on AI’s utility, and their confidence using AI tools. By focusing on first-year medical students at the University of Minnesota Medical School, this initiative seeks to inform future AI curriculum development and better prepare students for a future system where AI plays an integral role in their medical practice.

## Methods

This study was reviewed and approved by the University of Minnesota Medical School’s Institutional Review Board.

An interactive educational session was developed to introduce students to selected AI tools and their practical applications in medical education. The session was designed for first-year medical students at the University of Minnesota Medical School, aiming to enhance their understanding of AI technologies, their capabilities, and limitations in a variety of scenarios (Supplementary 1). The content included a foundational introduction to AI in medicine with discussions of benefits, harms, and limitations of AI tools such as ChatGPT (OpenAI), Co-Pilot (Microsoft), and Gemini (Google), and discussions on their potential role in improving medical knowledge, study efficacy, diagnostic accuracy, patient communication, and decision-making in clinical practice. The session was structured with a mix of lecture (55 min), live demonstration (10 min), hands-on activities (30 min) where students engaged directly with AI tools through a variety of prompts, and a large group discussion (5 min).

Student engagement activities involved:

- Case-based simulations requiring students to apply AI tools to solve clinical challenges.
- Using AI to create an individualized study plan.
- Generation of patient handouts improving patient education.
- Medical writing and research enhancement activities.
- Group discussions on the ethical considerations of AI in healthcare, focusing on bias, data privacy, and decision-making transparency.
- An interactive Q&A related to AI and medical education.

### Participants

A total of 180 of the 241(74%) first-year medical students from the University of Minnesota Medical School participated in the live session, recording provided to those unable to attend. Of 180 first-year medical students, 92 (51%) students filled out a post-session survey. Demographics characteristics of the participant cohort are described in Table 1.

**Table 1:** Demographic characteristics of medical student participants

Characteristic	Summary statistics (*n* = 92)
Age, median (range)	24 (21–35)
Male Sex, no. (%)	40 (43%)
Female Sex, no. (%)	51 (56%)
Other/Not Specified, no. (%)	1 (1%)
Self Identified Race, no. (%)	
Asian	13 (14%)
Black or African American	7 (8%)
Caucasian	64 (70%)
Hispanic	1 (1%)
2 + Races	4 (4%)
Other/Unknown	3 (3%)

This AI pilot session was a mandatory session part of the first-year’s “Becoming a Doctor” orientation week held over Zoom conferencing platform. Students were asked to evaluate the session’s content and delivery, their self-perceived understanding of AI tools, and their perspectives on AI’s potential strengths and limitations in healthcare settings. Specific metrics evaluated post-session perceived usefulness, confidence in using AI in education and/or clinical practice, and perceived ethical challenges. The data were analyzed using descriptive statistics to characterize the distribution of student responses. Importantly, because no pre-session was administered, results reflect post-session perceptions rather than measured changes from baseline.

### Tools Used

The session introduced a variety of AI tools relevant to medical education and clinical practice:

- ChatGPT (4o and 4o mini): Demonstrated as a versatile tool for a variety of tasks.
- Gemini (1.5 Flash and Pro): Mentioned as an alternative to ChatGPT and its similar varied uses.
- Co-Pilot: Mentioned as a versatile tool with a subscription purchased and provided by the institution to try out.

Within small breakout rooms, students engaged in hands-on exercises based upon a variety of use case examples provided as examples including creating a differential diagnosis list for common presenting concerns, search through the literature and produce an up to date summary, use case of writing enhancement, generation of a personalized study plan, simulate a clinical scenario, attempt interpretation of diagnostic images, ethical debate on the use of AI, and generation of patient education materials. These activities helped students gain practical experience with current AI technologies and understand their evolving roles and limitations in medical practice.

### Ethical Considerations

Throughout the lecture portion of this session, ethical discussions were integrated to address concerns about the use of AI in healthcare, particularly in terms of patient data privacy, bias, and the problematic role of AI in decision-making versus human oversight. Students were encouraged to reflect on these issues and contribute to the discussion on how AI can be responsibly integrated into medical practice.

### Data Analysis

Descriptive statistics were used to summarize the quantitative survey data, including frequencies and percentages for Likert-scale responses. For qualitative responses, thematic analysis was conducted to identify key themes related to student perceptions of AI. The analysis focused on recurring concerns and feedback about AI’s use in medical education. Statistical analyses were performed using R Statistical Software (Version 4.3.1; R Foundation for Statistical Computing, Vienna, Austria).

## Results

During the lecture component of this session a poll was given by Zoom showing that of those attending live and responding to the poll, 25% had used AI tools in the past, with the remaining 75% having no experience using AI tools.

**Fig. 1:**
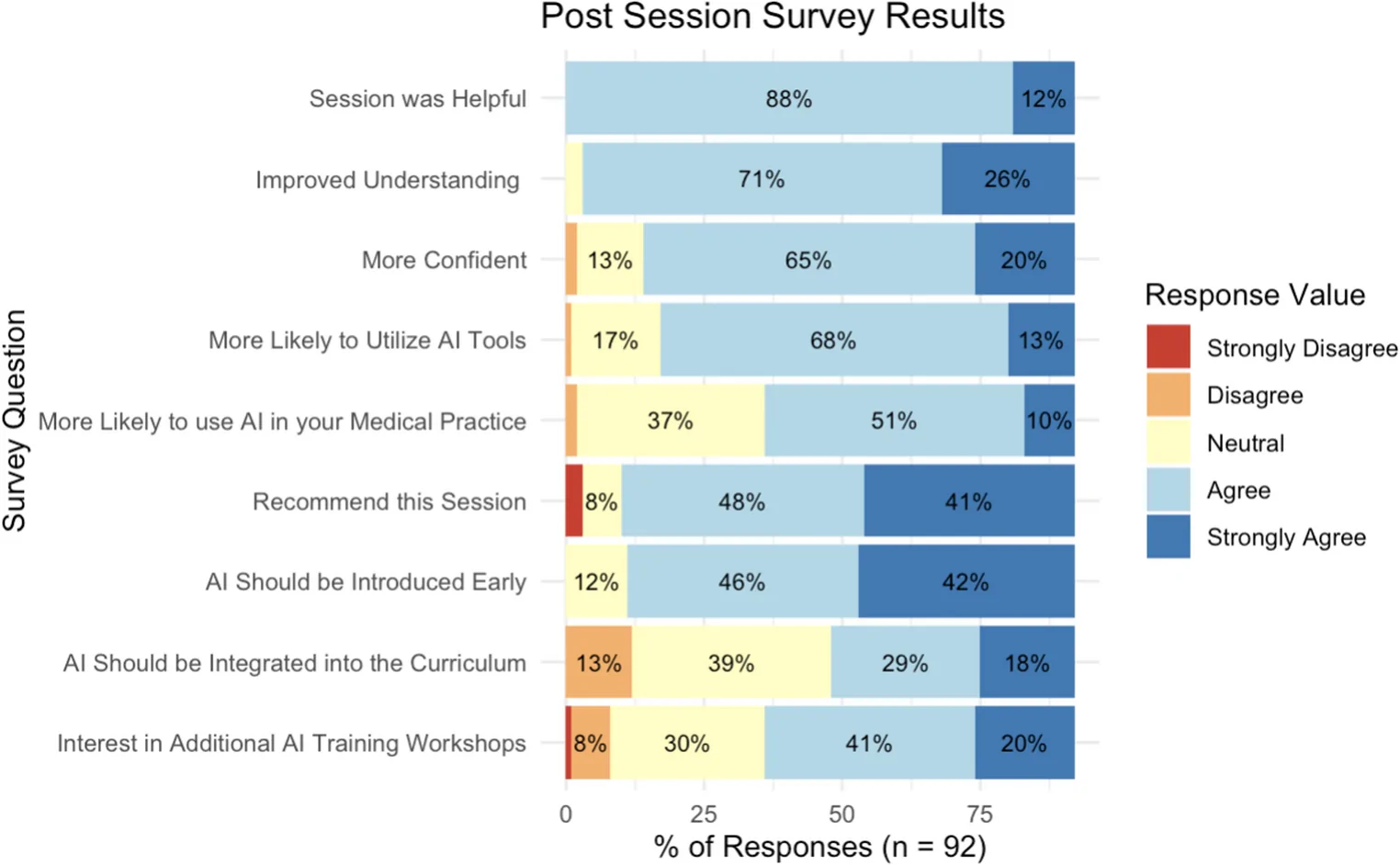
Stacked bar chart (Likert scale options) of responses of medical students (*n* = 92) to questions perceptions of an AI in Medical Education session, including usefulness, confidence, and likelihood of AI adoption

**Fig. 2:**
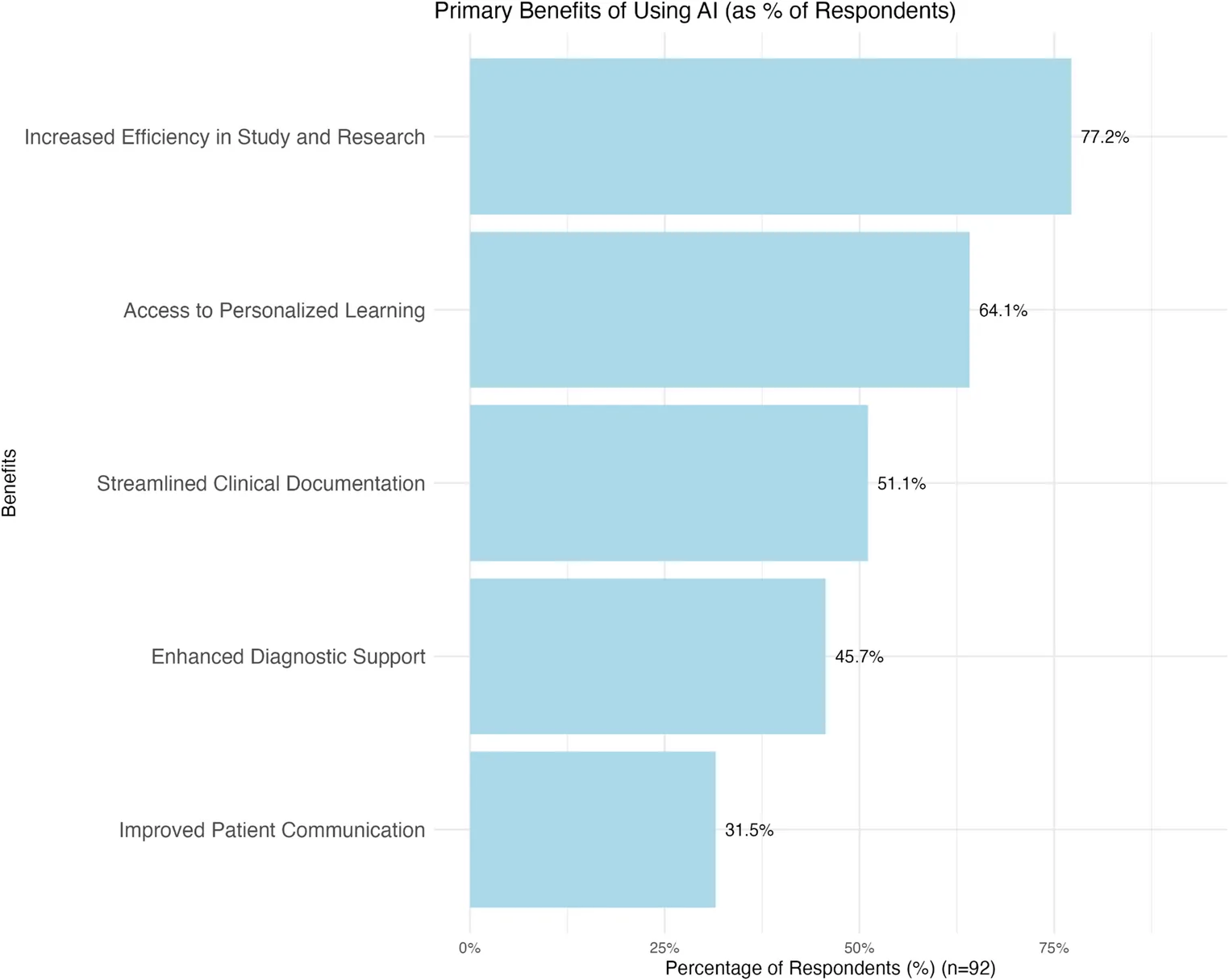
Bar chart of responses of medical students (*n* = 92) to potential benefits of AI in medicine and medical training

In the post-session survey, students expressed diverse views on the role of AI in medical education. The quantitative data from the Likert-scale responses (Figs. 1 and 2) illustrated that a significant portion of students recognized AI as a valuable tool. Many students reported that AI could enhance personalized learning (64.1%), with over half indicating that AI tools would help them tailor their educational experience. Additionally, a large number of respondents acknowledged the potential of AI in clinical decision-making, in streamlining clinical documentation (51.1%), and in providing diagnostic support (45.7%). Nearly all students viewed AI as an effective means to increase efficiency in their studies and research (77.2%), with the majority appreciating how AI could reduce the time spent on routine tasks like charting and research documentation.

Open-ended responses were also collected in the survey to gather further insight into students’ perceptions. A predominant theme emerged regarding students’ concerns about the accuracy and reliability of AI-generated information, particularly since they are new learners who may not recognize inaccurate information. Participants specifically documented instances where AI tools like ChatGPT “makes up research articles that aren’t real”, “presented us with fake publications”, and provided “unreliable articles most of the time” emphasizing that such information “definitely needs to be fact-checked.” Students consistently cautioned that “it’s best to double-check the information it gives,” noting that AI often “answers diagnosis questions wrong when you aren’t specific enough.”

Students also expressed apprehension regarding potential over-reliance on AI technology. One student “questioned if we might lose the ability to connect with our patients on a personal level if we became reliant on it,” while others were worried that convenient AI-generated summaries “may lead researchers to lose the ability to read high-level writing in research papers on their own.”

**Fig. 3:**
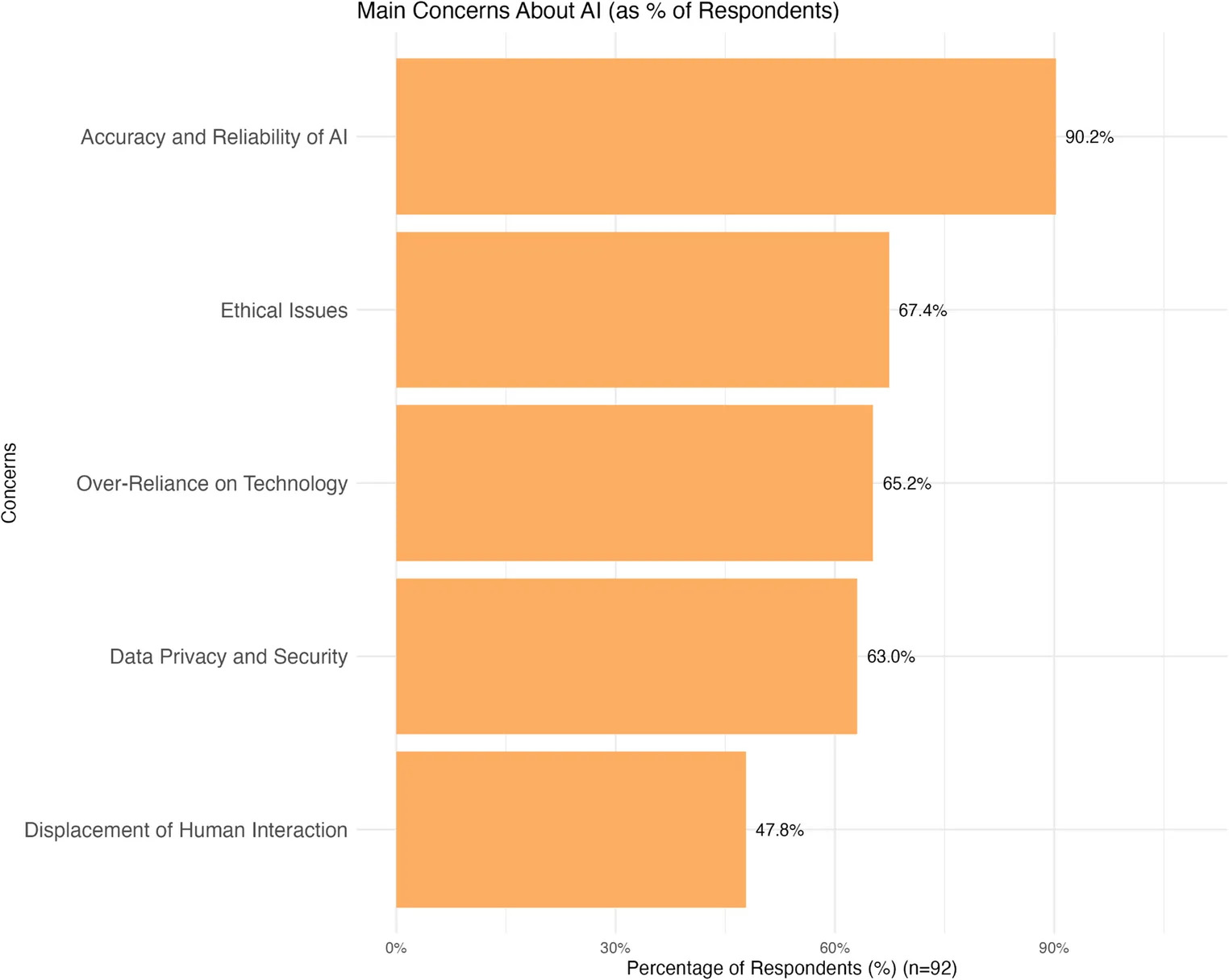
Bar chart of responses of medical students (*n* = 92) to the concerns of AI in medicine and medical training

The possibility of biases within AI systems was another prevalent concern, with respondents highlighting the risks associated with perpetuating existing healthcare disparities. Ethical issues and data privacy were also frequently mentioned, as students emphasized the importance of protecting sensitive patient data in the context of AI-driven tools **(**Fig. 3**).**

## Discussion

Introducing AI technology into early medical education has the potential to improve learning outcomes and offers a structured, equitable environment in which students can explore emerging technologies. The post-session survey data from this introductory session along with prior work, positively favor the early incorporation of AI content within undergraduate medical studies.

Importantly, this session extends the existing literature not only by adding to previously reported attitudinal trends, but also by grounding an AI introductory session in established pedagogical theory, documenting specific patterns of student uncertainty, and providing detailed curricular blueprint for iterative improvement.

According to student survey responses, AI was perceived as beneficial for enabling personalized learning, improving efficiency in studying and research, and streamlining documentation. The session’s structure which incorporated elements of didactic instruction, live demonstration, hands-on application, and reflective group discussion, mirrors Kolb’s experiential learning cycle and reflects constructivist principle of active and social learning [9, 10]. This design may have contributed to the favorable perceptions observed, as students not only saw how AI could be used, but also had the ability to engage with it in various prompts/situations and discuss both the method and results with their peers. Further observations from our post-sessions survey found that students felt more confident in applying AI tools following the session, suggesting that an introductory session may provide an equitable opportunity for students to explore how AI may impact their study and medical career. This is critical as at the time 75% of the students indicated during the session that they never used AI tools before. While the proportion of students with prior experience with AI tools will certainly increase through the years, providing an introductory session allows for an equitable baseline for all students [15].

Conversely, student post-session responses also highlighted strong concern for accuracy and reliability (90.2%), followed by ethical issues (67.4%), over-reliance on technology (65.2%), data privacy and security (63.0%), and displacement of human interaction (47.8%). Recent studies have elucidated some of these concerns with excessive reliance on AI in medical decision making could hinder the development of clinical judgement and critical thinking [16]. A recent study measuring electroencephalographic (EEG) waves found that writers assisted by LLM’s engaged in fewer executive-language networks and struggled to recall their own texts, reflecting the trade-off between deep-learning and technology convenience [17]. These findings highlight the importance of AI curricula early in undergraduate medical education, while having great potential to enhance student learning, can also have a deleterious effect as well.

These findings indicate that students are aware of both the practical limitations and broader implications of AI integration in healthcare systems and medical education. Their concerns about information reliability reflect an understanding of the current technical constraints of AI systems, while their apprehensions about over-dependence demonstrate recognition of the importance of maintaining fundamental clinical competencies. The student perspectives documented here suggest that successful AI integration in healthcare education may require approaches that emphasize validation protocols, preserve essential skill development, and maintain human elements central to effective patient care.

## Limitations

This study has several important limitations that should be considered when interpreting its findings. First and most significantly, no pre-session assessment was administered, meaning the study can only report post-session perceptions rather than measured changes or improvements from baseline. All claims regarding student confidence, understanding, and attitudes should be interpreted as self-reported cross-sectional perceptions captured at a single time point following the intervention, not as evidence of demonstrated change attributable to the session. Future iterations should incorporate matched pre- and post-session assessments to enable comparison and more robust evaluation of educational impact.

Second, the study relied exclusively on descriptive statistics, which are appropriate for an exploratory pilot but limit the analytical depth of the findings. Future studies targeting subgroup analyses, such as comparing perceptions of students with prior AI experience (25%) to those without (75%) could provide additional insight into whether baseline familiarity moderates post-session attitudes. Given how the data was structured, such comparisons were not feasible in the present analysis.

Third, the session was delivered via Zoom to capture both Duluth and Twin-Cities campuses, which may limit student engagement relative to an in-person format. The impact of the in-person versus virtual delivery has not been assessed.

Lastly, the post-session survey response rate of 51% (92 of 180 attendees) introduces the possibility of response bias, as students who chose to complete the survey may have been more engaged with or favorable toward the session content than non-respondents. The extent to which the sample is representative of the full cohort is unknown.

## Conclusion

This pilot study provides preliminary descriptive data on first-year medical students’ perceptions following a novel introductory AI education session. The post-survey findings suggest that pedagogical theory based, structured, early exposure AI tools may support foundational AI literacy for medical students.

Post-session survey responses reflected a majority of students recognizing the potential value for AI in personalized learning, didactic and research efficiency, and clinical documentation. Yet, concurrently, students demonstrated nuanced awareness of AI’s limitations expressing concern for accuracy, bias, ethical concerns, data privacy, and the risk of over-reliance. This coexistence of caution and enthusiasm suggest that an introductory session has the potential to elicit important consideration for AI use, even if it does not fundamentally shift confidence or attitudes across learners.

### Future Directions

The orientation session will continue for future student cohorts at the University of Minnesota Medical School. However, based upon the results of this study, including minimal student background in using AI, more demonstration of basic AI prompting and iterative generation will be incorporated, more time will be given for student practice with using AI in virtual small breakout rooms, and more time for Q&A with large group debrief and discussion will be planned. The student cohort was quite thoughtful regarding the limitations and harms of using AI so this will again be emphasized with recommendations for students on best practices of using AI as a teammate and a catalyst for production, and when to consult a faculty member. Existing instruction in evidence appraisal and uncertainty management as already incorporated into medical education, should expand to encompass AI-related decision-making. These skills ought to be expanded to include AI as a tool in the physician’s armamentarium.

To build on the momentum of this pilot program, future sessions and data collection will include longitudinal follow-up to assess the long-term effects of AI education on knowledge, attitudes, and behavior. The longitudinal course called “clinical skills” in our curriculum, tasked with educating students on essential clinical competencies, will be introducing dedicated AI sessions for pre-clinical medical students in future years with more direct consideration of AI use in future practice including separated and spaced sessions related to:

1. Clinical and healthcare specific uses including differential diagnosis, documentation, answering clinical questions, prior authorization documentation, and other clinical tasks.
2. Non-clinical uses such as for time management and organization tasks, email drafts or professional communication, research and project management frameworks, coaching, and others.
3. In depth discussion of the Ethics of AI including but not limited to future role as a physician and physician responsibilities, bias and impact on underserved populations, environmental considerations, and others.

Plans include collaboration with clerkship directors to introduce AI-integration discussions relating to clinical practice, learn from what students have seen in their rotations, and have a more complete understanding of the rapidly evolving integration of AI into healthcare to inform future pre-clinical sessions’ content and considerations.

Investigating the integration of AI into these additional aspects of medical education could provide deeper insights into AI’s value and the development of standardized AI curricula incorporating meaningful ethical principles, crucial in equipping future physicians with the necessary skills and judgment to navigate an increasingly AI-integrated healthcare environment.
